# Substantial Changes of Gaseous Pollutants and Health Effects During the COVID‐19 Lockdown Period Across China

**DOI:** 10.1029/2021GH000408

**Published:** 2021-05-24

**Authors:** Chaohao Ling, Yongfei Li

**Affiliations:** ^1^ State Key Laboratory of Lake Science and Environment Nanjing Institute of Geography and Limnology Chinese Academy of Sciences Nanjing China; ^2^ University of Chinese Academy of Sciences Beijing China; ^3^ Hunan Provincial Key Laboratory of Ecological Tourism College of Tourism & Management Project Jishou University Zhangjiajie China

**Keywords:** COVID‐19, health effect, NO2, random forest

## Abstract

The human movement and economic activities have been drastically reduced due to the Coronavirus Disease 2019 (COVID‐19) outbreak, leading to the sharp decreases of pollutant emissions and remarkable air quality improvement. Nevertheless, however, the changes of gaseous pollutant concentrations and health effects across China during the COVID‐19 lockdown period remained poorly understood. Here, a random forest model was applied to assess the impact of COVID‐19 lockdown on pollutant concentrations and potential health effects. The results suggested that estimated NO_2_, SO_2_, and CO concentrations in China during January 23–March 31, 2020 decreased by 13.68%, 25.71%, and 7.42%, respectively compared with the same periods in 2018–2019. Nonetheless, the predicted 8‐h O_3_ concentrations across China suffered from 1.29% increases during this period. The avoided premature all‐cause, cardiovascular disease (CVD), respiratory disease (RD), and chronic obstructive pulmonary disease (COPD) mortalities induced by NO_2_ decrease during COVID‐19 lockdown period reached 3,954 (3,076–4,832), 635 (468–801), 612 (459–765), and 920 (653–1,186) cases. However, the increases of all‐cause, CVD, RD, and COPD mortalities due to O_3_ increase during COVID‐19 lockdown period achieved 462 (250–674), 79 (29–129), 40 (−25–105), and 52 (−34–138) cases. The natural experiment demonstrated the drastic emission reduction measures could significantly decrease the NO_2_, SO_2_, and CO concentrations, while they significantly elevated the O_3_ concentration. It is highly imperative to propose more coordinated air pollution control strategies to control O_3_ pollution.

## Key Points

- The impacts of Coronavirus Disease 2019 lockdown on gaseous pollutants across China were investigated
- The 8‐h O_3_ level displayed slight increase, while other pollutants showed dramatic decreases
- COVID‐19 saved 3,954 lives due to NO_2_ decrease, but it claimed 462 lives linked with O_3_ increase

## 1 Introduction

As a highly contagious respiratory virus, Coronavirus Disease 2019 (COVID‐19) was first reported in Wuhan during the first half December 2019 and then spread to more than 200 countries around the world (Li et al., 2020; Zu et al., 2020). Until August 22, more than 22.81 million confirmed COVID‐19 cases and 790 thousand deaths worldwide (https://www.worldometers.info/). Due to its high infectious, many governments have to raise a series of control measures to restrict human activities and prevent the spread of epidemic. On January 23, a day before the Lunar New Year of 2020, Chinese government imposed a lockdown in Wuhan and significantly restricted citizen mobility throughout the country (Bauwens et al., 2020). The blocked roads, checkpoints, as well as the closure of industries and restaurants across the whole China forced many people to stay at home (Chang et al., 2020). These associated reduction of business, industry and traffic inevitably resulted in the decreases of pollutant emissions, and might improve the local air quality (Baldasano, 2020; Kroll et al., 2020). Although some previous studies have assessed the response of air quality improvement to emission reduction during the periods of APEC Blue and Parade Blue (Guo et al., 2016; Xu et al., 2017), most of these events only focused on a urban or regional scale. In contrast, COVID‐19 lockdown provided an unprecedented chance to estimate the short‐term effects of economic activity counterfactual to “business as usual” at a national scale.

Recently, some researches have quantified the short‐term trends of gaseous pollutants both from space and surface perspectives (He et al., 2020; Lian et al., 2020). C. Fan et al., (2020) observed that both of NO_2_ and CO columns in China displayed significant decreases during COVID‐19 lockdown period based on satellite products. Later on, Shi and Brasseur (2020) also confirmed that the surface NO_2_ and CO concentrations over China decreased by 55% and 23%, respectively. The substantial decreases of pollutant concentrations certainly resulted in the increases the health benefits. Bray et al., (2021) observed that global NO_2_ column based on satellite (ozone monitoring instrument (OMI) on Aura) reduced by approximately 9.19% and 9.57% during March–April. Chen et al., (2020) applied the observation data to estimate that about 8,911 NO_2_‐related deaths could be avoided during the COVID‐19 outbreak period. Unfortunately, the use of satellite product or surface observation alone did not accurately reflect the effect of COVID‐19 lockdown on the air quality alleviation. It was well known that the column concentrations generally represented the total concentrations of gaseous pollutants in the troposphere even the stratosphere (McLinden et al., 2014), which were not entirely derived from surface anthropogenic emissions. Thus, some researchers used the ground‐level observation data to assess the impact of COVID‐19 on air quality. Mahato et al., (2020) found that both of NO_2_ and CO in Delhi, India also showed considerable declines during lockdown. However, each isolated site only possessed limited spatial representative area (0.25–16.25 km^2^) and the trend analysis based on these monitoring sites alone might overestimate the decrease trend because most of these sites were located in the urban areas and these areas were more sensitive to the emission reduction compared with the rural regions (Li, Cui, et al., 2020; Shi et al., 2018). Moreover, the monitoring sites were unevenly distributed over China, and some key regions (e.g., Hubei province) showed scarce monitoring sites, which could significantly increase the probability of exposure misclassification and the uncertainty of assessment result (Li, Cui, et al., 2020; Li, Zhao, et al., 2020). Thus, it was highly imperative to combine the surface observation data and satellite product to develop an empirical model to fill the gaps lack of monitoring sites and then to accurately assess the short‐term variations and health effects of gaseous pollutants during COVID‐19 lockdown period across China.

Here, we employed the random forest (RF) model to predict the gridded NO_2_, SO_2_, CO, and 8‐h O_3_ concentrations across China during January 23, 2020–March 31 in 2018–2020. Then, the difference of pollutant concentrations during COVID‐19 lockdown period and those during the same periods in 2018 and 2019 were quantified. Finally, the health benefits (costs) of NO_2_ and O_3_ during this period were determined.

## 2 Materials and Methods

### 2.1 Ground‐Level Observation Datasets

The daily NO_2_, SO_2_, CO, and 8‐h O_3_ datasets during January 23, 2020–March 31, 2020 across China were downloaded from the website of Ministry of Ecology and Environment of the People's Republic of China (http://www.cnemc.cn/en/). Meanwhile, these gaseous pollutant datasets during the same periods in 2018 and 2019 were also obtained from the website to compare the annual variation and to assess the effect of COVID‐19 lockdown. This period was selected to assess the impact of COVID‐19 lockdown on air quality since most of the residents have been forced to stay at home. After March 31, many provinces formulated some policies to resume production though the epidemic was not over.

The ground‐level observation network has expanded to 1,641 monitoring sites covering 336 cities in 31 provinces (autonomous region, municipalities) across China, all of which were depicted in Figures S1 and S2. All of these monitoring sites were designed as a mixture of urban, suburban, and background sites. These monitoring sites suffered from unevenly distributed across the entire China. Most of these sites focused on East China, while the West China possessed relatively scarce monitoring sites especially in the Tibetan Plateau. The data quality in all of the sites were assured on the basis of HJ 630–2011 specifications.

### 2.2 Input Variables

The tropospheric NO_2_ column density, total SO_2_ column, and total O_3_ column (spatial resolution: 0.25°) were collected from ozone monitoring instrument (OMI) level‐3 product onboard the Aura satellite to estimate the surface NO_2_, SO_2_, and 8‐h O_3_ concentrations, respectively. The retrievals of surface CO mixing ratios obtained from measurements of pollution in the troposphere were used as the key variable to predict the surface CO concentrations across China. The gaseous pollutant columns derived from OMI with cloud radiance fraction >0.5, terrain reflectivity >30%, and solar zenith angles >85 must be removed. In addition, the cross‐track pixels frequently influenced by row anomaly should be deleted. The retrievals of CO mixing ratios were resampled to 0.25° grids using area‐weighted average method.

Apart from these satellite products, some meteorological data and geographical covariates should be added into the model (Table S1). The meteorological data including 2 m dewpoint temperature (D_2m_), evaporation (E), mean boundary layer dissipation (Mbld), surface pressure, T_2m_, total precipitation, 10 m U wind component (U_10_), and 10 m V wind component (V_10_) (spatial resolution: 0.25°) during 2018–2020 were obtained from European Centre for medium‐range weather forecasts (ECMWF). The 30‐m resolution elevation data set was collected from geographical and spatial data cloud. The data of population density (1 km resolution) were obtained from the China Resource and Environmental Science Data Center . Additionally, the land use data with 30 m resolution (e.g., waters, grassland, urban, forest, and agricultural land) were also incorporated into the model.

### 2.3 Modeling Methodology

The RF approach produced a large amount of decision trees based on independent bootstrap samples. Each node of decision tree was split depending on the best result with the traversal of all the variables which were randomly selected at that node. At last, the lowest out‐of‐bag error was selected to assure the optimal model. The model has been widely applied to estimate the air pollutant concentrations and accurately captured nonlinear and high‐order interactions between the predictors and dependent variables. The detailed algorithm of RF model is summarized as follows (Wei, Huang, et al., 2019; Wei, Li, et al., 2019):

(1) $$f\left(x\right)=\sum_{z=1}^{Z}c_{z}I\left(x\in M_{z}\right)$$

(2) $$\overset{\Delta }{c_{z}}=\text{mean}\left(y_{i}|x_{i}\in M_{z}\right)$$

(3) $$Z_{1}\left(m,n\right)=\left\{X|X_{j}\leq n\right\}\&Z_{2}\left(m,n\right)=\left\{X|X_{j}>n\right\}$$

(4) $$\min_{m,n}\left[\min\sum_{M_{1}\left(m,n\right)}{\left(y-c_{1}\right)}^{2}+\min\sum_{M_{2}\left(m,n\right)}{\left(y-c_{2}\right)}^{2}\right]$$

(5) $$\overset{\Delta }{c_{1}}=\text{mean}\left(y_{i}|x_{i}\in M_{1}\left(m,n\right)\right)\&\overset{\Delta }{c_{2}}=\text{mean}\left(y_{i}|x_{i}\in M_{2}\left(m,n\right)\right)$$

where (*x*_i_, *y*_i_) denotes the sample for *i* = 1, 2, …, *N* in *M* regions (*M*_1_, *M*_2_, …, *M*_z_), I represents the weight of the tree branch, *L* is the branch of each decision tree, *c*_*m*_ represents the response to the model, $\overset{\Delta }{c_{z}}$ denotes the best value, *m* represents the feature variable, c_1_ represents the average of left branch, while c_2_ denotes the average of right branch. *n* is the split point.

In our study, the RF model was applied to estimate the daily concentrations of gaseous pollutants during January 23–March 31 in 2018, 2019, and 2020. To evaluate the modeling performance of RF approach, sample‐based 10‐fold cross‐validation technique was utilized to test the predictive power. Besides, the by‐year cross‐validation method was applied to validate the generalization ability of the model. The determination coefficient (*R* ^2^), root mean square error (RMSE), and mean absolute error (MAE) were selected as the key statistical indicators to quantitatively assess the model performance.

### 2.4 The Mortality Estimates During January 23, 2020–March 31, 2018–2020

The premature mortality due to excessive NO_2_ and O_3_ exposure was calculated based on the following equation (Li, Zhao, et al., 2020):

(6) $$M=y_{0}\left(1-1/\exp\left[\text{ER}\times \left(C-C_{0}\right)\right]\right)\times \text{Pop}$$

where *M* denotes the premature mortality due to excessive NO_2_ and O_3_ exposures; ER represents the exposure‐response coefficient (Tables S2 and S3); *y*_*0*_ represents baseline mortality of a specific disease (Table S4); *C* denotes the estimated 8‐h O_3_ level, *C*_*0*_ denotes the threshold value without health risk (NO_2_: 40 μg/m3, 8‐h O_3_: 100 μg/m^3^); Pop is the exposure population in each cell. In our study, the mortalities attributable to all‐cause disease, cardiovascular disease ozone monitoring instrumentCVD, respiratory disease (RD), and chronic obstructive pulmonary disease (COPD) were calculated based on Equation 1. The health benefits (costs) during COVID‐19 lockdown period were estimated based on the minus of mortalities between 2020 and 2018–2019.

## 3 Results and Discussion

### 3.1 Model Evaluation

The satellite data, meteorological factors, elevation, land use types, and other geographical covariates were applied to estimate the gridded NO_2_, SO_2_, CO, and 8‐h O_3_ concentrations across China during January 23–March 31 in 2018, 2019, and 2020 using RF model. As shown in Figure S3, the CV *R* ^2^ values for NO_2_ estimates in 2018, 2019, 2020, and 2018–2020 were 0.70, 0.74, 0.53, and 0.70, respectively. Both of RMSE and MAE were in the order of 2018 (10.32 and 7.53 μg/m^3^) > 2018–2020 (9.99 and 7.21 μg/m^3^) > 2020 (9.97 and 7.15 μg/m^3^) > 2019 (9.61 and 6.90 μg/m^3^). The CV R^2^ values for SO_2_ and CO estimates showed the similar annual variations to NO_2_ estimation (Figure S4 and S5), following the order of 2018 (SO_2_ and CO: 0.66 and 0.66) > 2019 (0.66 and 0.63) > 2018–2020 (0.64 and 0.59) > 2020 (0.47 and 0.38). RMSE (MAE) for SO_2_ and CO showed the highest (SO_2_: 10.58 (6.16 μg/m^3^) and CO: 0.28 (0.20 mg/m^3^)) and lowest ones (SO_2_: 7.23 (4.34 μg/m^3^) and CO: 0.31 (0.20 mg/m^3^)) in 2018 and 2020, respectively. The 8‐h O_3_ estimation displayed the highest *R* ^2^ value in 2019 (0.80), followed by 2018 (0.79), 2018–2020 (0.73), and the lowest value in 2020 (0.60) (Figure S6). However, RMSE and MAE for 8‐h O_3_ estimation displayed the highest values in 2020 (15.94 and 11.10 μg/m^3^).

The predictive accuracy of RF model exhibited significantly yearly difference. In general, the CV R^2^ values for pollutant estimates in 2018 and 2019 were significantly higher than 2020. It was assumed that the response of satellite products (column concentrations) to sharp changes of surface pollutant concentrations during the COVID‐19 lockdown period might be not very sensitive. Both of RMSE and MAE for most pollutants except O_3_ showed the highest values in 2018, followed by 2019 and 2020, which might be attributable to relatively higher concentrations of gaseous pollutants in 2018. On the contrary, both of RMSE and MAE showed the highest values in 2020 because the surface O_3_ concentrations still suffered from persistent increases across China in recent years (Liu et al., 2020).

**Figure 1:**
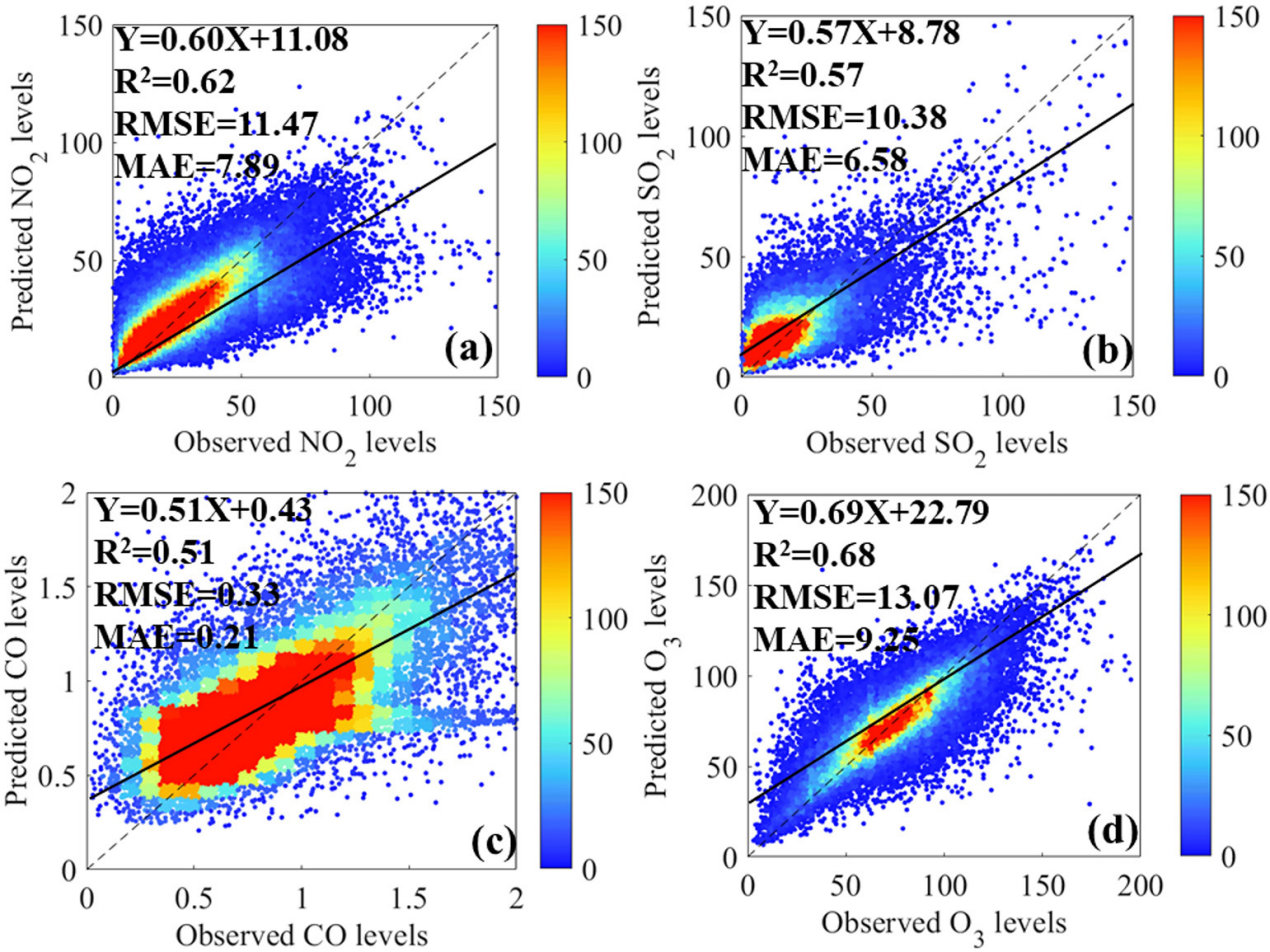
Density scatterplots of the by‐year cross‐validation results for the NO_2_, SO_2_, CO, and O_3_ estimates. The linear regression relationships between observed NO_2_, SO_2_, CO, and O_3_ levels and corresponding predicted values are also given in each panel. The black solid line represent the optimal fitting line through the data points. The black dashed line is the diagonal line.

Overall, the predictive performances for all of the pollutant estimation during 2018–2020 were robust, while the transferability of this model was still remained unknown. Therefore, the by‐year CV was applied to test the model's transferability in order to ensure the robustness of this model. As shown in Figure 1, the by‐year *R* ^2^ values of NO_2_, SO_2_, CO, and 8‐h O_3_ estimates across China were 0.62, 0.57, 0.51, and 0.68, respectively. These R^2^ values were only slightly lower than the CV *R* ^2^ values of training models, and both of RMSE and MAE for by‐year CV results were in good agreement with the training models. All of these results confirmed that the RF model could be employed to analyze the temporal changes and health benefits caused by COVID‐19 lockdown.

### 3.2 The Dramatic Changes of Gaseous Pollutant Concentrations During COVID‐19 Lockdown Period

**Figure 2:**
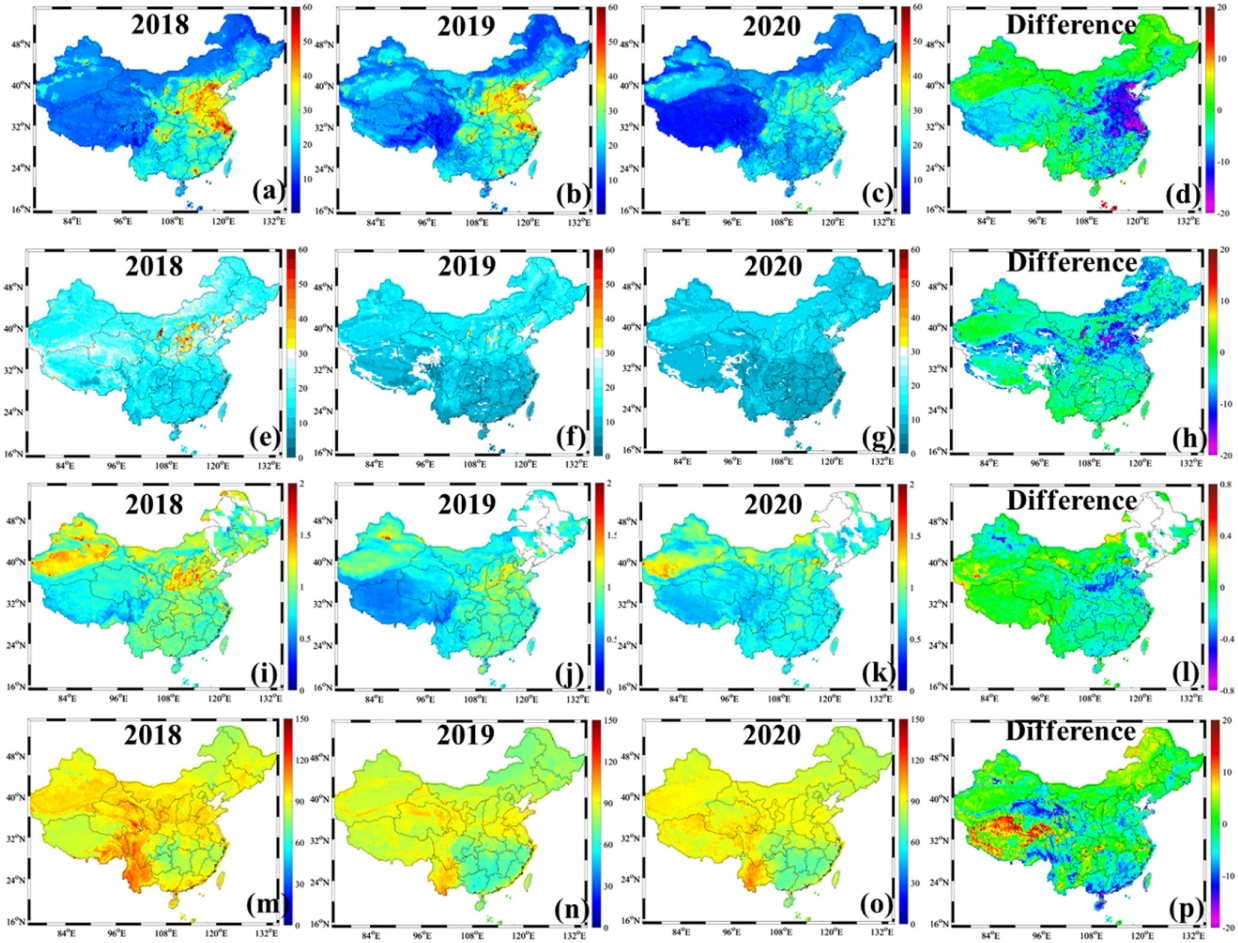
The spatiotemporal variations of NO_2_, SO_2_, CO, and 8‐h O_3_ concentrations during January 23–March 31 (COVID‐19 outbreak) in 2018 (a, e, i, and m), 2019 (b, f, j, and n), and 2020 (c, g, k, and o). The difference of NO_2_, SO_2_, CO, and 8‐h O_3_ concentrations during COVID‐19 outbreak in 2020 and ones during the same period in 2018–2019.

As shown in Figure 2, the estimated NO_2_, SO_2_, and CO concentrations in China during January 23–March 31, 2020 decreased by 13.68%, 25.71%, and 7.42%, respectively compared with the same periods in 2018–2019 (Figure S7–S12). However, the predicted 8‐h O_3_ concentrations across China suffered from 1.29% increases during the COVID‐19 lockdown period (Figure S13). The dramatic decreases of NO_2_ and SO_2_ concentrations in China during this period was attributable to the substantial emission reduction of NO_x_ and SO_2_ associated with the shutdown of industries and reduction of vehicular transportation and domestic flights (>70%) (Chang et al., 2020). Miyazaki et al. (2020) also verified that both of the NO_x_ and SO_2_ emissions across China in 2020 decreased by 36% compared with 2015. Compared with NO_2_ and SO_2_, the CO concentrations seems to show the slight variation during COVID‐10 lockdown. It was assumed that CO was regarded as a product of residential combustion and power generation (H. Fan et al., 2020; Wang et al., 2019), and the home quarantine enhanced residential burning (heating and cooking), which might offset the decrease of industrial emission. In contrast, the surface O_3_ concentration across China displayed slight increase during this period. It was assumed that the aerosol decrease might promote the O_3_ increase because the aerosols scavenge HO_2_ and NO_x_ radicals that otherwise would produce O_3_ (Shi & Brasseur, 2020). Tie et al., (2005) reported that the loss of the HO_2_ radical on the surface of sulphate particles significantly prohibited the O_3_ formation, which explained the inverse relationship between NO_2_ and O_3_ concentrations.

The concentration changes of these gaseous pollutants response to COVID‐19 lockdown varied greatly at the spatial scale. For all of the gaseous pollutants, the dramatic changes focused on East China including BTH, YRD, PRD, and Wuhan. Compared with 2018–2019, the NO_2_, SO_2_, and CO concentrations in Wuhan decreased by 41.11%, 25.71%, and 15.46% in 2020, respectively. Following Wuhan, the NO_2_ concentrations in BTH, YRD, and PRD decreased by 26.99%, 34.84%, and 24.40%, respectively. The SO_2_ concentrations in BTH, YRD, and PRD reduced by 39.40%, 38.76%, and 22.66%, respectively. The CO concentrations in these regions decreased by 16.63%, 15.53%, and 13.81%, respectively. The 8‐h O_3_ concentrations in BTH, YRD, PRD, and Wuhan increased by 0.89%, 2.86%,−3.61%, and 3.76%, respectively. Among all of these regions, the NO_2_, SO_2_, and CO concentrations in Wuhan exhibited the most striking decrease owing to the earliest and most drastic measures to reduce people's exposure to the COVID‐19. Following Wuhan, both of BTH and YRD suffered from remarkable air pollution alleviation. YRD experienced more remarkable NO_2_ decrease, while BTH exhibited more dramatic SO_2_ decrease. It was supposed that more of the industrial points such as coal‐fired power plants and cement industries were located on BTH (Qi et al., 2017). The sudden outbreak of COVID‐19 caused the shutdown of these industries, which facilitated the SO_2_ decrease. Nevertheless, YRD suffered from frequent NO_3_^−^ pollution events due to the high loadings of NO_x_ emission, and thus the COVID‐19 lockdown triggered the rapid decrease of NO_2_ concentration (Sun et al., 2019; Yao et al., 2019). The O_3_ changes in different regions were inversely related with the NO_2_ variations. Monks et al., (2015) revealed that nitric oxide (NO) emitted into the atmosphere converted a large fraction of O_3_ into NO_2_ when NO emission was sufficient.

**Figure 3:**
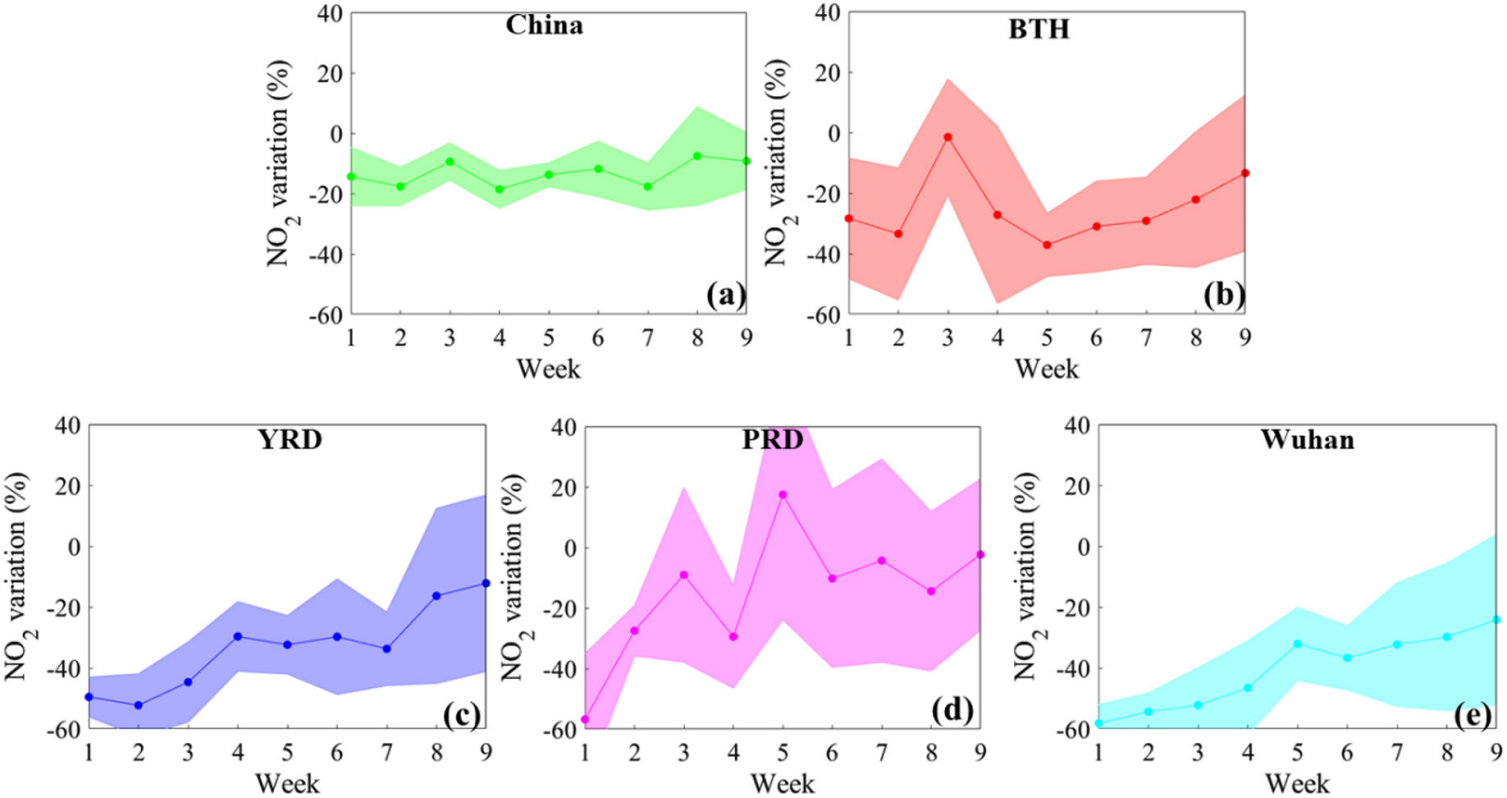
The ambient NO_2_ variation ratios in China (a), BTH (b), YRD (c), PRD (d), and Wuhan (e) during the COVID‐19 outbreak compared with the same period during 2018–2019. The positive value denotes the NO_2_ increase, while the negative one represents the NO_2_ decrease.

In order to further reveal the impact of COVID‐19 lockdown on gaseous pollutant changes, the temporal variability of the difference between 2020 and 2018–2019 were shown in Figure 3. We can find that the weekly variability of NO_2_ concentration in some major regions (e.g., Wuhan) totally displayed the gradual increases during the COVID‐19 lockdown period, while the weekly variability of NO_2_ level across China was not pronounced. It was supposed that some western provinces generally possessed less pollution emissions compared with the developed regions of East China (Azimi et al., 2018; Sun et al., 2018; van der A et al., 2017), and thus the response of air quality improvement to emission reduction was not significant. Wuhan suffered from the sharp decrease of NO_2_ concentration since the first week (−58.19%) because Chinese government first imposed a lockdown in Wuhan. After the lockdown in Wuhan, the lockdown policies were expanded to many megacities of China (C. Fan et al., 2020), and thus the sharp decreases of NO_2_ concentrations in BTH, YRD, and PRD were lagged behind about one week. After four weeks of COVID‐19 outbreak, the decreases of NO_2_ levels have been significantly shrunken because many cities began to resume production and the anthropogenic emissions began to increase (Chang et al., 2020). In PRD, the NO_2_ concentrations in late March 2020 returned to the same levels as 2018–2019. The weekly variability of SO_2_ and CO displayed the similar characteristics to NO_2_, while the duration of CO decline was longer than NO_2_ and SO_2_ (Figures S11 and S12). In contrast to these pollutants, the 8‐h O_3_ concentration showed the decreasing trend during COVID‐19 lockdown except the sporadic week (The fifth week in PRD) due to the unfavorable meteorological conditions. Based on the original data, the fifth week in PRD was characterized with the static weather including low wind speed (2.4 m/s), which caused the higher ozone concentration during this week.

### 3.3 The Health Benefits (Costs) Associated With COVID‐19 Lockdown

The substantial changes of air pollutant concentrations during COVID‐19 period generally plays an important role on the human health, which could be estimated based on population, baseline incidence rates for specific outcomes, and epidemiological exposure‐response functions. Owing to the short window of the COVID‐19 lockdown, we only estimated the short‐term health benefits (costs) associated with NO_2_ and 8‐h O_3_ exposure. In our study, we estimated the avoided premature mortalities and derived from CVD, RD, and COPD and the total mortalities during January 23–March 31, 2020 and the same periods during 2018–2019. The difference of mortalities were regarded as the health benefits (costs) during COVID‐19 lockdown. As shown in Table 1, the avoided all‐cause, CVD, RD, and COPD mortalities induced by NO_2_ decrease during COVID‐19 lockdown period reached 3,954 (3,076–4,832), 635 (468–801), 612 (459–765), and 920 (653–1,186) cases. Among the major developed regions across China, YRD (all‐cause mortality: 1,738 (1,353–2,122)) and BTH (all‐cause mortality: 990 [771–1,209]) showed the higher health benefits because the NO_2_ concentrations in these regions experienced rapid decreases. Although Wuhan suffered from remarkable NO_2_ decrease, the all‐cause mortality in this city was still lower than those in some megacities due to the relatively few population. Nonetheless, the mortalities derived from O_3_ exposure showed the slight increases in most regions across China. The increases of all‐cause, CVD, RD, and COPD mortalities due to O_3_ increase during COVID‐19 lockdown period reached 462 (250–674), 79 (29–129), 40 (−25–105), and 52 (−34–138) cases. The spatial characteristics of mortalities due to O_3_ increase were in good agreement with those induced by NO_2_ decrease. Both of YRD and BTH suffered from the higher health costs because of the O_3_ increase. In PRD, the mortalities induced by O_3_ exposure during COVID‐19 lockdown period still showed the decreasing trend because the local NO_2_ and PM_2.5_ concentrations did not show significant decreases compared with BTH and YRD. Based on the estimates, COVID‐19 lockdown saved 3,954 lives due to the NO_2_ decrease, while it led to about 462 mortalities owing to the O_3_ increase. Overall, the air pollution declines response to the COVID‐19 lockdown might play an important role on the disease transmission and health care system. It should be noted that our estimates of health benefits (costs) still suffers from uncertainties. First of all, the exposure‐response coefficient was obtained from previous references (Chen et al., 2018), and the parameter might vary during different study periods. Besides, the estimates of pollutant concentrations also show some uncertainties, which increase the errors of health effect assessment.

**Table 1:** Avoided Premature Deaths (95% Confidence Interval) Triggered by NO_2_ and O_3_ Variation During the Lockdown Period

Air pollutant	Study region	All‐cause	Cardiovascular disease	Respiratory disease	COPD
NO_2_	China	3,954 (3,076–4,832)	635 (468–801)	612 (459–765)	920 (653–1,186)
	BTH	990 (771–1,209)	171 (126–215)	165 (124–205)	247 (176–318)
	YRD	1,738 (1,353–2,122)	299 (220–377)	288 (216–359)	432 (307–556)
	PRD	324 (252–396)	72 (53–91)	70 (52–87)	105 (74–135)
	Wuhan	168 (131–205)	26 (19–33)	25 (19–32)	38 (27–49)
O_3_	China	−462 (−674–−250)	−79 (−129–−29)	−40 (‐105–25)	−52 (‐138–34)
	BTH	−24 (−35–−13)	−4 (−7–−1)	−2 (‐6–1)	−3 (‐8–2)
	YRD	−101 (−147–−55)	−19 (−30–−8)	−9 (‐25–6)	−12 (‐32–8)
	PRD	42 (23–61)	10 (4–16)	5 (‐3–13)	7 (‐4–17)
	Wuhan	−17 (−9–−25)	−3 (−1–−5)	−2 (‐4–1)	−2 (‐5–1)

*Notes*. The positive value indicates the health benefits during COVID‐19 lockdown, while the negative one suggests the health costs.

Abbreviation: COPD, chronic obstructive pulmonary disease.

## 4 Conclusions and Implications

The unprecedented steps performed to stop the transmission of COVID‐19 plays an important role on the air pollution alleviation. The estimated NO_2_, SO_2_, and CO concentrations in China during COVID‐19 lockdown decreased by 13.68%, 25.71%, and 7.42%, respectively compared with the same periods in 2018–2019, while the predicted 8‐h O_3_ concentrations across China experienced 1.29% increases during this period. The dramatic decreases of NO_2_, SO_2_, and CO concentrations in China during COVID‐19 lockdown was attributable to the substantial emission reduction associated with the shutdown of industries and reduction of vehicular transportation and domestic flights (>70%). The surprise increases of O_3_ concentrations was attributable to the aerosol decrease, which generally scavenge HO_2_ and NO_x_ radicals that otherwise would produce O_3_.

The substantial changes of air pollutant concentrations during COVID‐19 lockdown period inevitably influence the human health. The avoided premature all‐cause, CVD, RD, and COPD mortalities induced by NO_2_ decrease during COVID‐19 lockdown period reached 3,954 (3,076–4,832), 635 (468–801), 612 (459–765), and 920 (653–1,186) cases. However, the increases of all‐cause, CVD, RD, and COPD mortalities due to O_3_ increase during COVID‐19 lockdown period achieved 462 (250–674), 79 (29–129), 40 (−25–105), and 52 (−34–138) cases. Among all of the developed regions across China, both of YRD and BTH suffered from the higher health benefits (costs) during the COVID‐19 period. Overall, COVID‐19 lockdown saved 3,954 lives due to the NO_2_ decrease, while it led to about 462 mortalities owing to the O_3_ increase in China.

The natural experiment shed light upon that the stringent lockdown measures significantly decreased the concentrations of NO_2_, SO_2_, and CO concentrations because the human movement and economic activities have been strictly restricted. However, the 8‐h O_3_ concentrations did not show remarkable decrease even displayed slight increases in most regions across China. The result indicated that the reduction of industrial emission and vehicle emission were beneficial the dramatic decrease of CO and NO_2_ concentrations. Thus, the ultralow emission measures and oil quality improvement should be further implemented. In addition, the emissions of VOCs and carbonaceous aerosols should be also constrained in order to control the elevation of O_3_ concentration. Moreover, the coordinated air pollution control strategies of PM_2.5_ and O_3_ are needed because excessive PM_2.5_ emission reduction might promote O_3_ production.

## Conflict of Interest

The authors declare that they have no competing interests.

## Supporting information

Supporting Information S1

## Data Availability

The land use types and population data are provided by Data Center for Resources and Environmental Sciences, Chinese Academy of Sciences (RESDC) (https://www.aqistudy.cn/historydata/), in Chinese.
