# Supplementary material for: Substantial Changes of Gaseous Pollutants and Health Effects During the COVID‐19 Lockdown Period Across China
Source: Geohealth. 2021 May 24;5(5):e2021GH000408. doi: 10.1029/2021GH000408 (PMC8144698; doi:10.1029/2021GH000408)
Supplement: Supplementary file 1 — Supporting Information S1 [file GH2-5-e2021GH000408-s001.doc]

**Substantial changes of gaseous pollutants and health effects during the COVID-19 lockdown period across China**

Chaohao Ling^1,2*^, Yongfei Li ^3^

^1^ State Key Laboratory of Lake Science and Environment, Nanjing Institute of Geography and Limnology, Chinese Academy of Sciences, Nanjing, 210008, China;

^2^ University of Chinese Academy of Sciences, Beijing, 100049, China;

^3^Hunan Provincial Key Laboratory of Ecological Tourism, College of Tourism & Management project, Jishou University, 427000, Zhangjiajie, China

*** Corresponding author.** Chaohao Ling (chhling@niglas.ac.cn)

**Figure S1.** The geographical locations of major study regions across China including BTH (green), YRD (yellow), and PRD (purple).


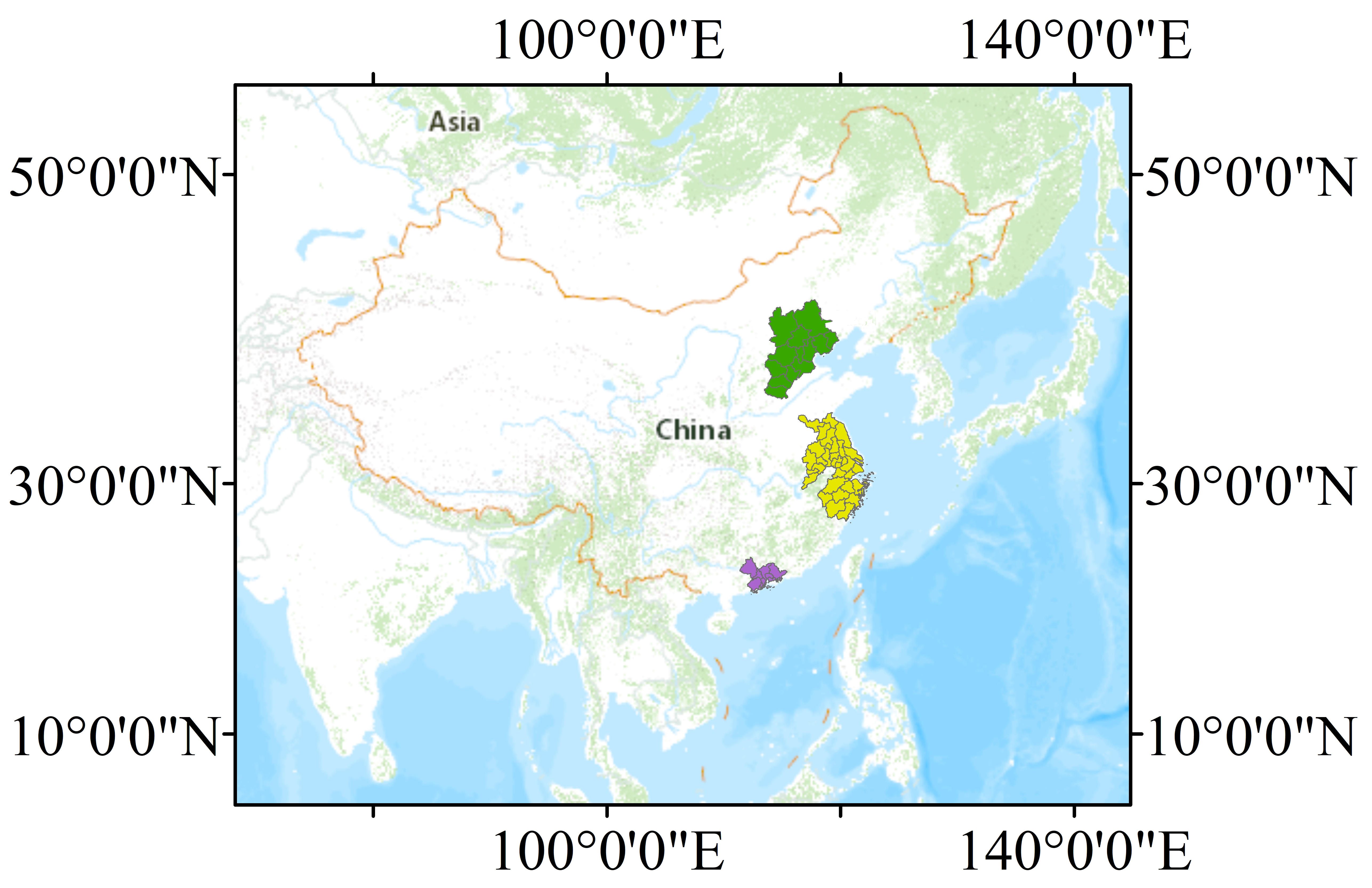


**Figure S2.** The spatial distributions of ground-level monitoring sites for gaseous pollutants (SO_2_, NO_2_, CO, and O_3_) involved in model fitting and validation. Red circles denote the ground-level sites during 2018-2020. The colormap reflects the elevation variation across China.


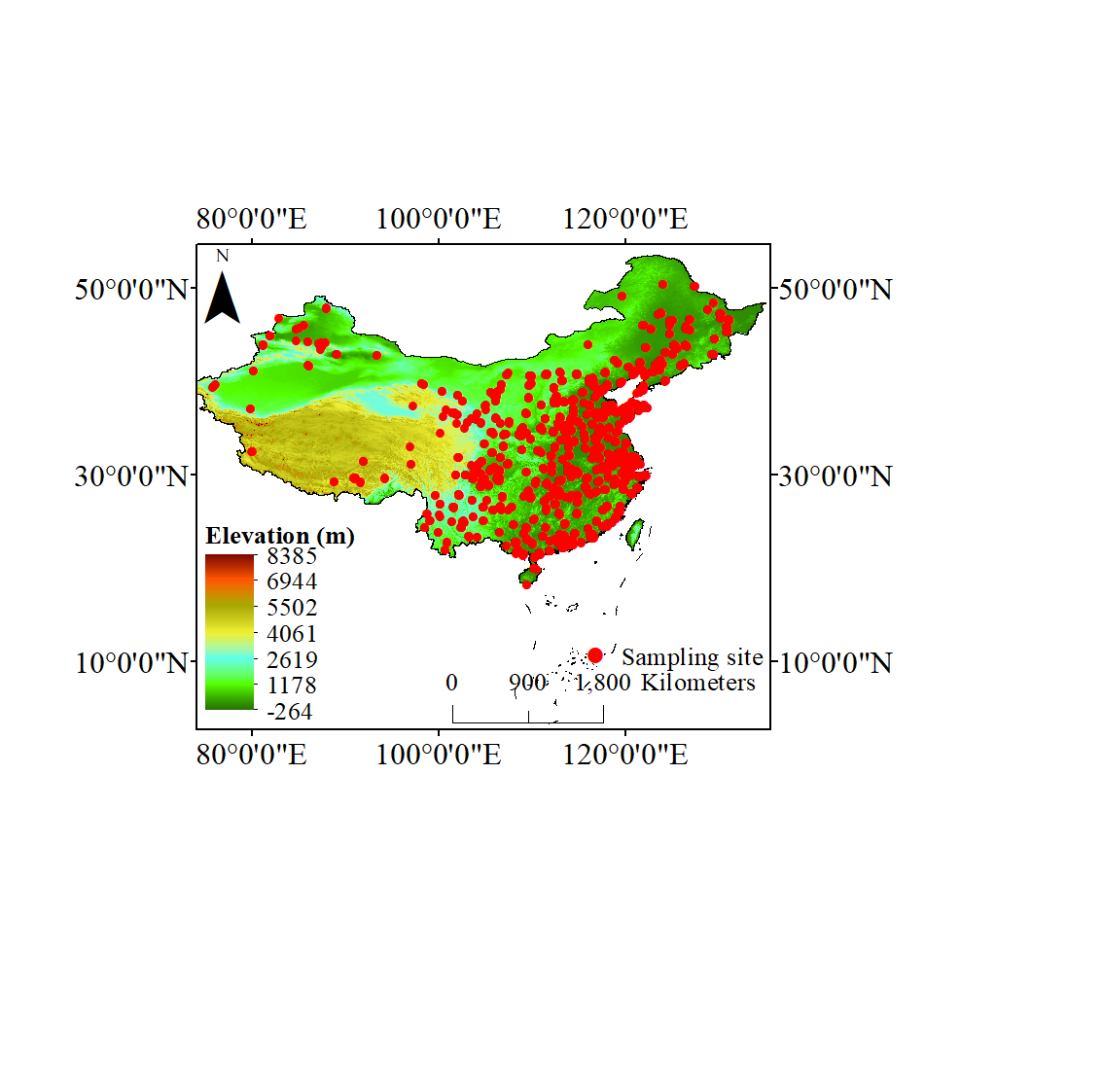


**Figure S3.** Density scatterplots of cross-validation (CV) predictive results for NO_2_ estimates in 2018 (a), 2019 (b), 2020 (c), and 2018-2020 (d) using RF model. The linear regression relationship between observed values and predicted values is also given in each panel. The black full lines represent the optimal fitting lines through the data points. The black dashed lines denote the diagonal line.


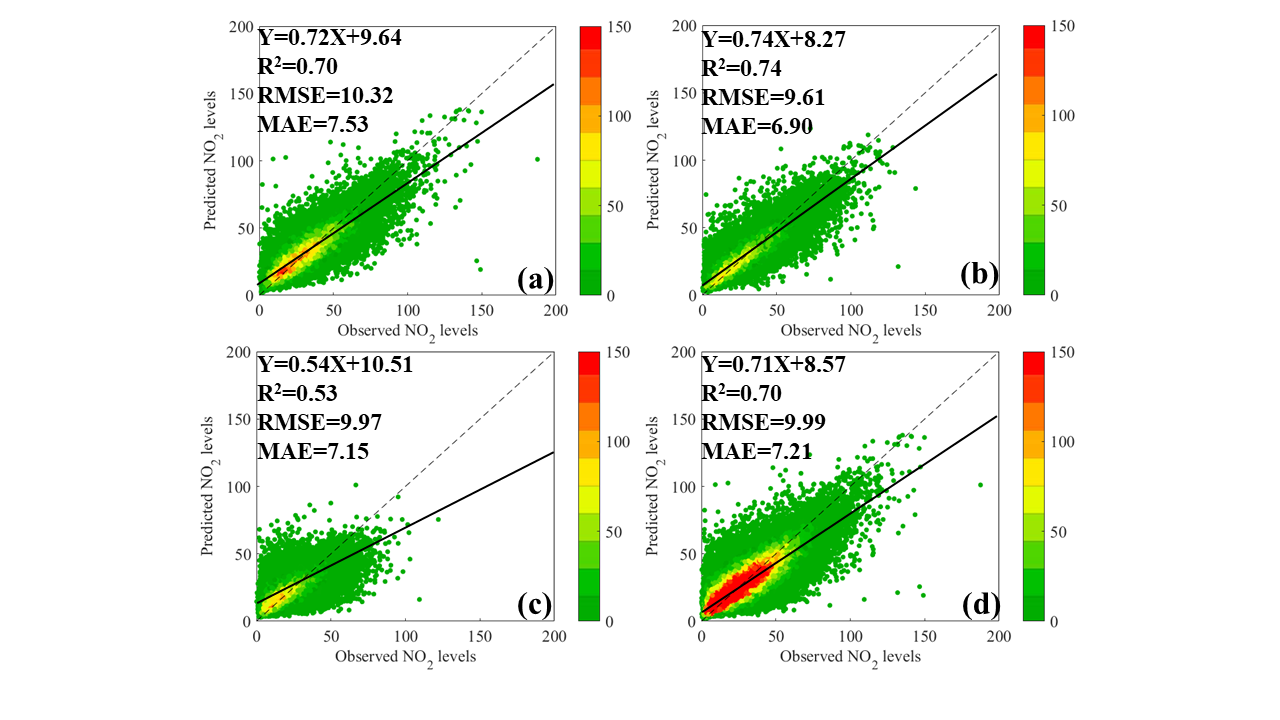


**Figure S4.** Density scatterplots of cross-validation (CV) predictive results for SO_2_ estimates in 2018 (a), 2019 (b), 2020 (c), and 2018-2020 (d) using RF model. The linear regression relationship between observed values and predicted values is also given in each panel. The black full lines represent the optimal fitting lines through the data points. The black dashed lines denote the diagonal line.

**
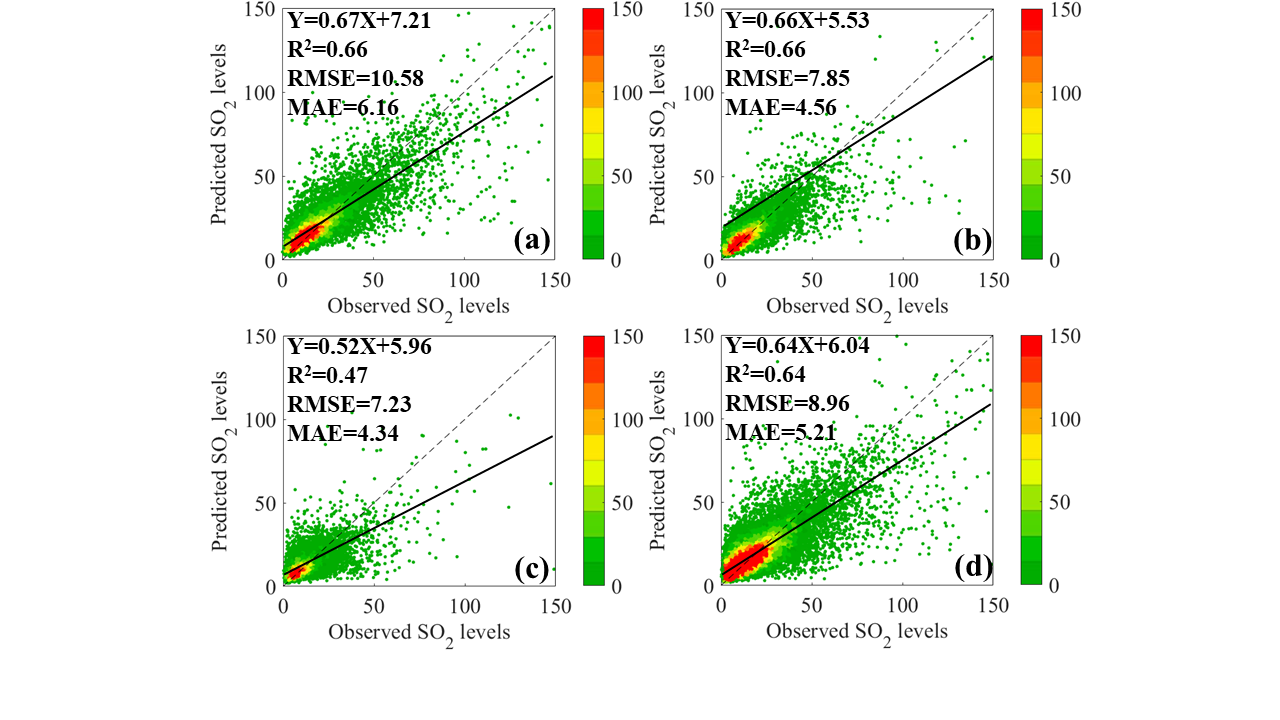
**

**Figure S5.** Density scatterplots of cross-validation (CV) predictive results for CO estimates in 2018 (a), 2019 (b), 2020 (c), and 2018-2020 (d) using RF model. The linear regression relationship between observed values and predicted values is also given in each panel. The black full lines represent the optimal fitting lines through the data points. The black dashed lines denote the diagonal line.

**
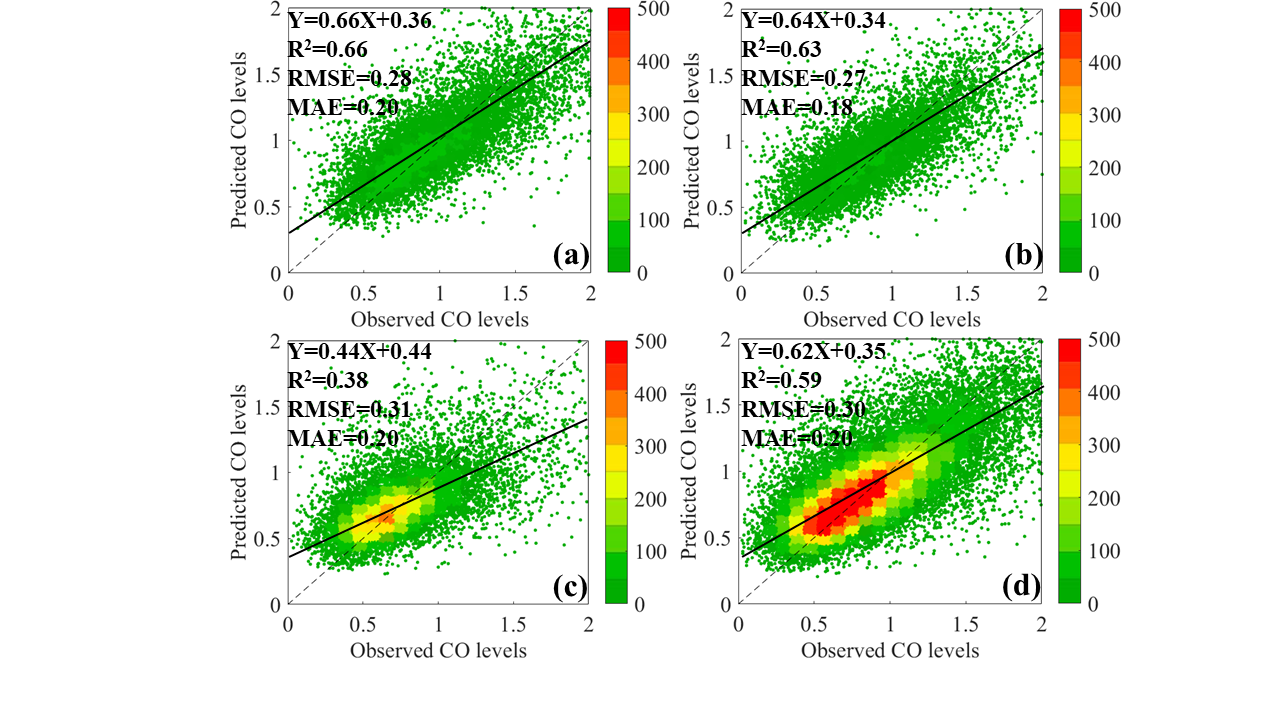
**

**Figure S6.** Density scatterplots of cross-validation (CV) predictive results for O_3_ estimates in 2018 (a), 2019 (b), 2020 (c), and 2018-2020 (d) using RF model. The linear regression relationship between observed values and predicted values is also given in each panel. The black full lines represent the optimal fitting lines through the data points. The black dashed lines denote the diagonal line.

**
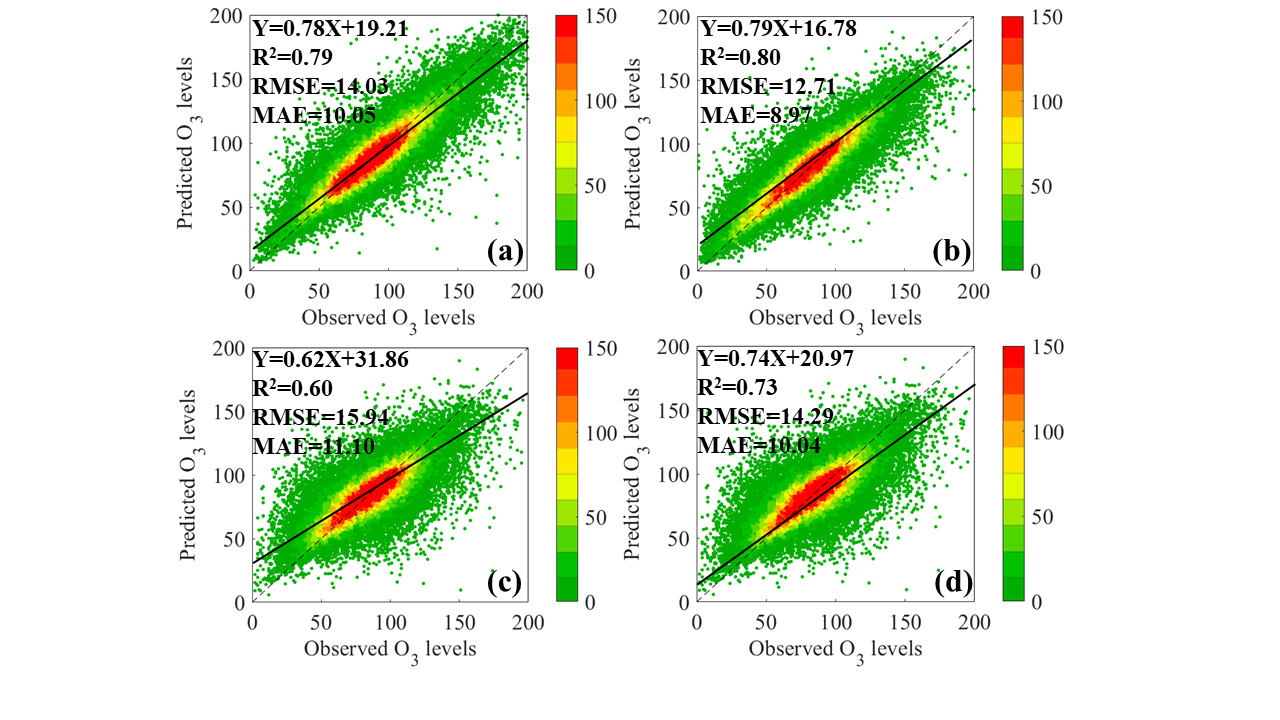
**

**Figure S7.** The weekly variations of NO_2_ concentrations in China (a), BTH (b), YRD (c), RPD (d), and Wuhan (e) during COVID-19 outbreak in 2020 and the same periods in 2018 and 2019.


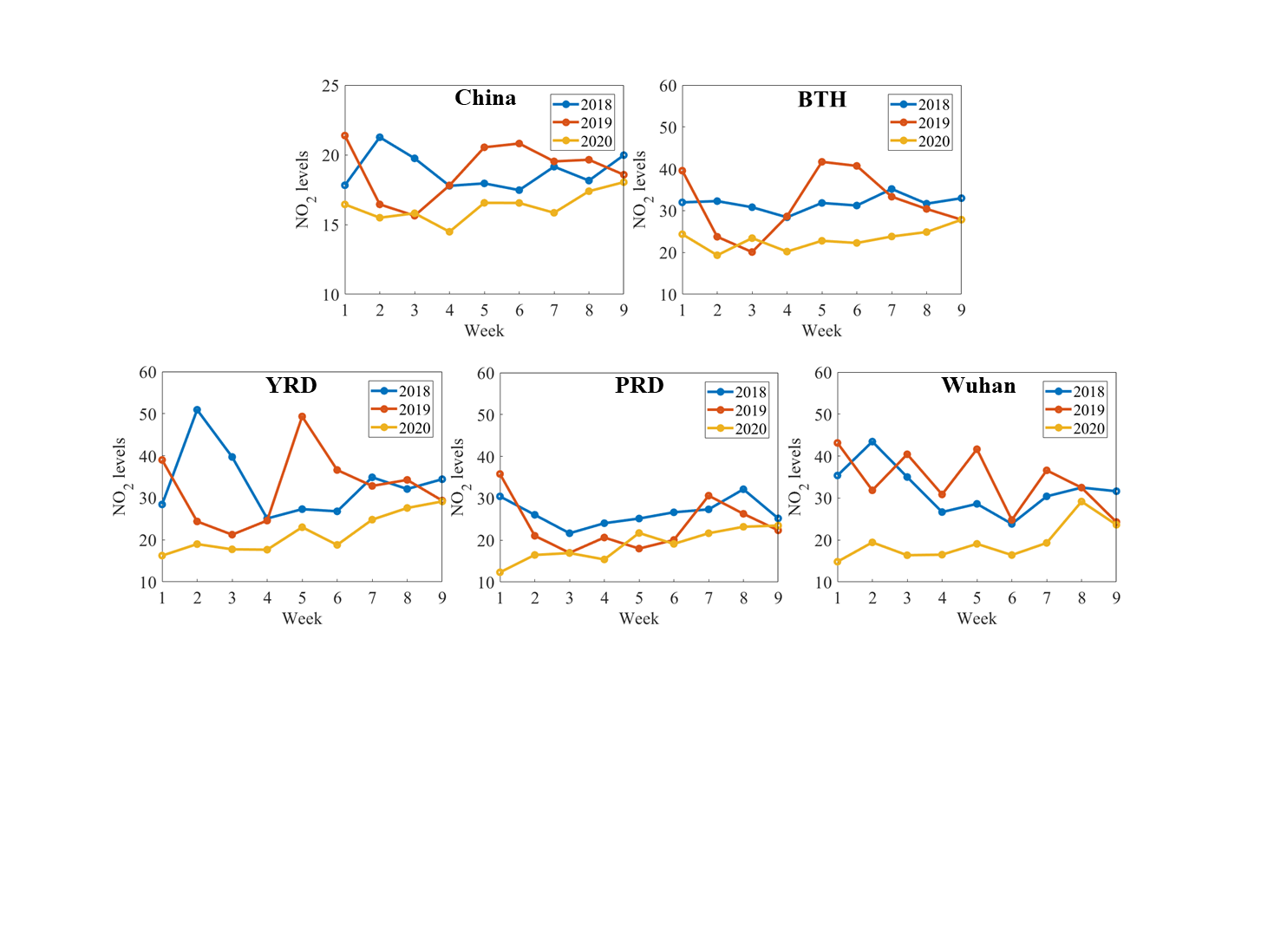


**Figure S8.** The weekly variations of SO_2_ concentrations in China (a), BTH (b), YRD (c), RPD (d), and Wuhan (e) during COVID-19 outbreak in 2020 and the same periods in 2018 and 2019.

**
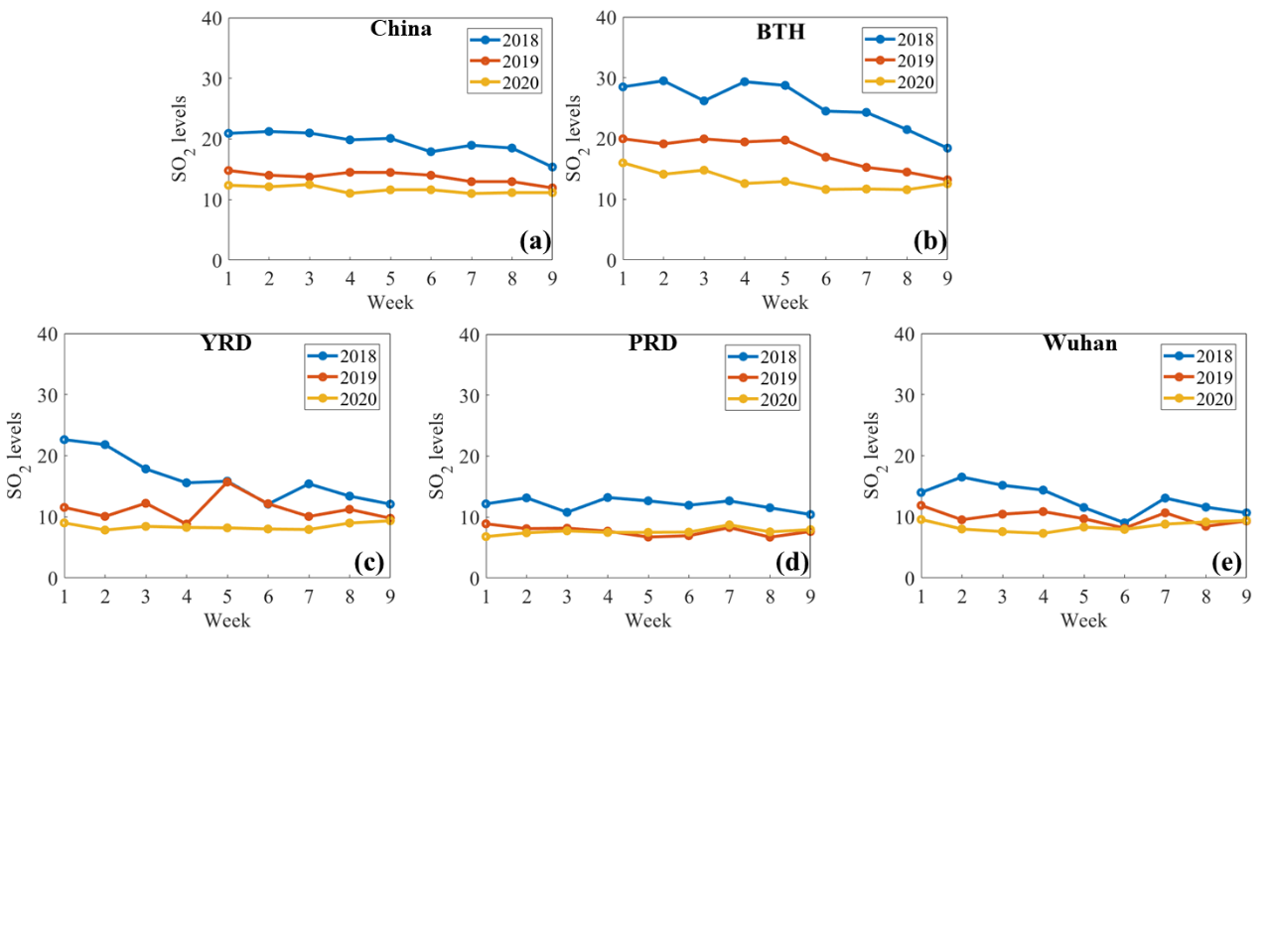
**

**Figure S9.** The weekly variations of CO concentrations in China (a), BTH (b), YRD (c), RPD (d), and Wuhan (e) during COVID-19 outbreak in 2020 and the same periods in 2018 and 2019.

**
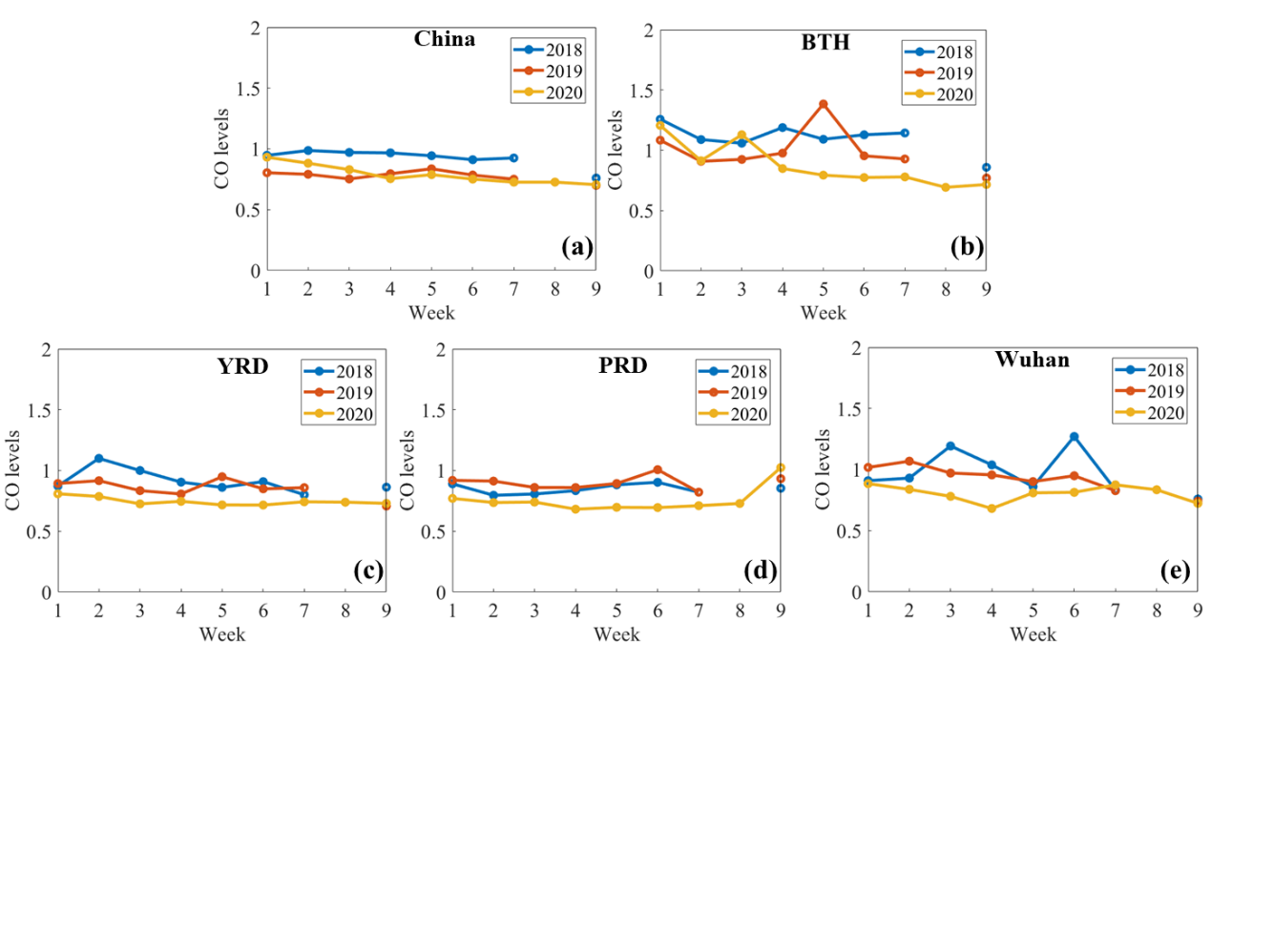
**

**Figure S10.** The weekly variations of 8-h O_3_ concentrations in China (a), BTH (b), YRD (c), RPD (d), and Wuhan (e) during COVID-19 outbreak in 2020 and the same periods in 2018 and 2019.

**
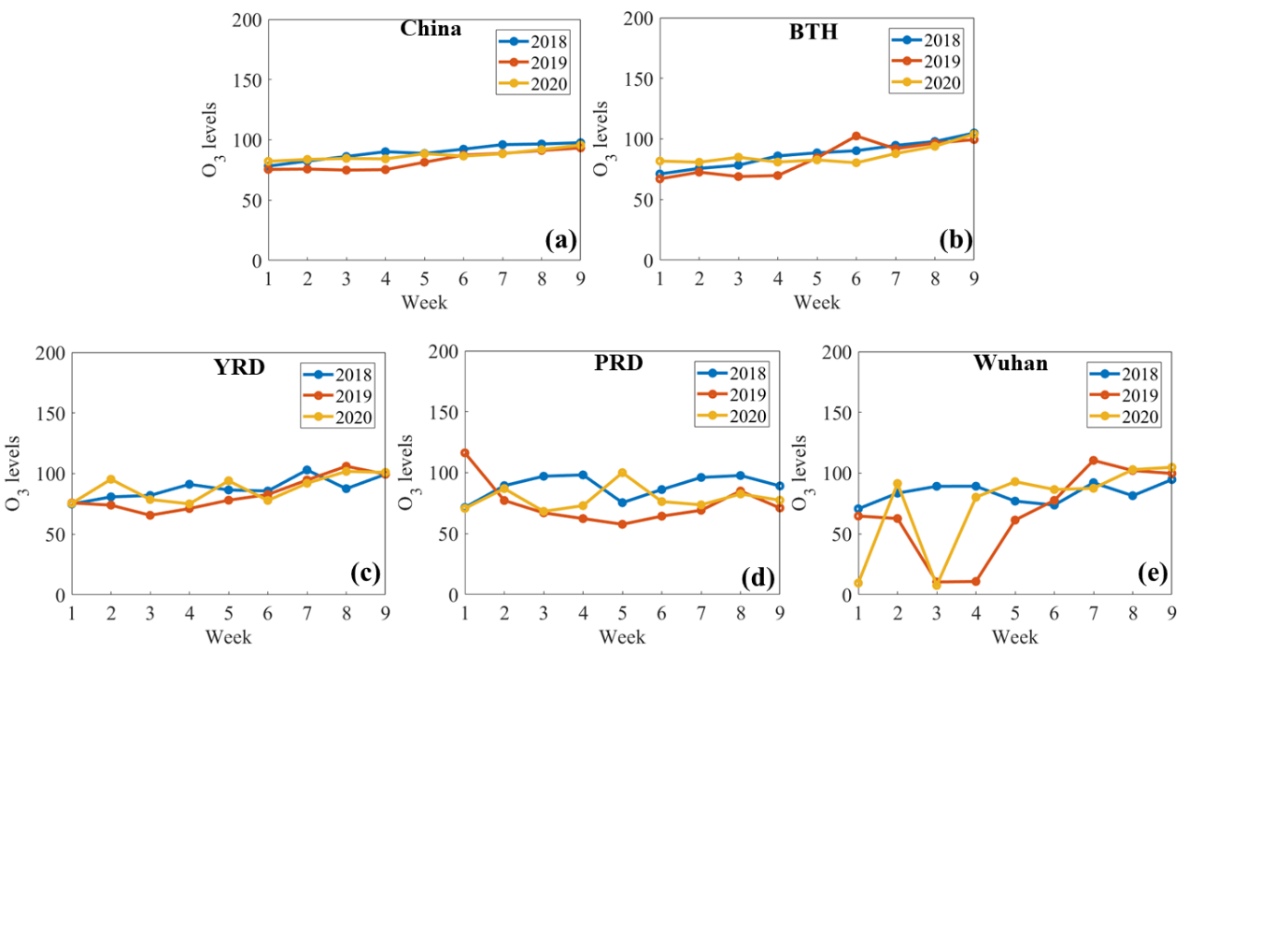
**

**Figure S11.** The ambient SO_2_ variation ratios in China (a), BTH (b), YRD (c), PRD (d), and Wuhan (e) during the COVID-19 outbreak compared with the same period during 2018-2019.

**
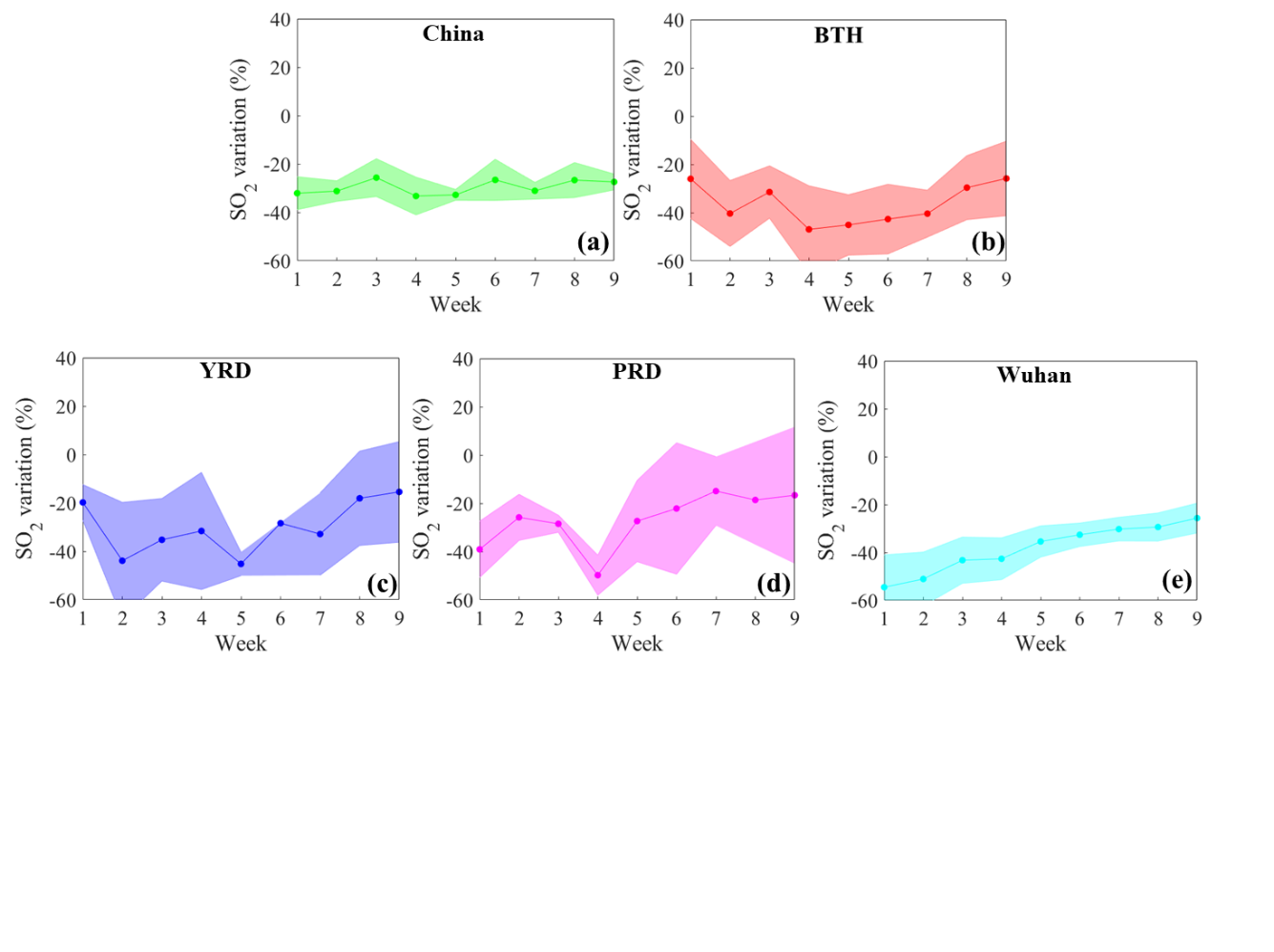
**

**Figure S12.** The ambient CO variation ratios in China (a), BTH (b), YRD (c), PRD (d), and Wuhan (e) during the COVID-19 outbreak compared with the same period during 2018-2019.

**
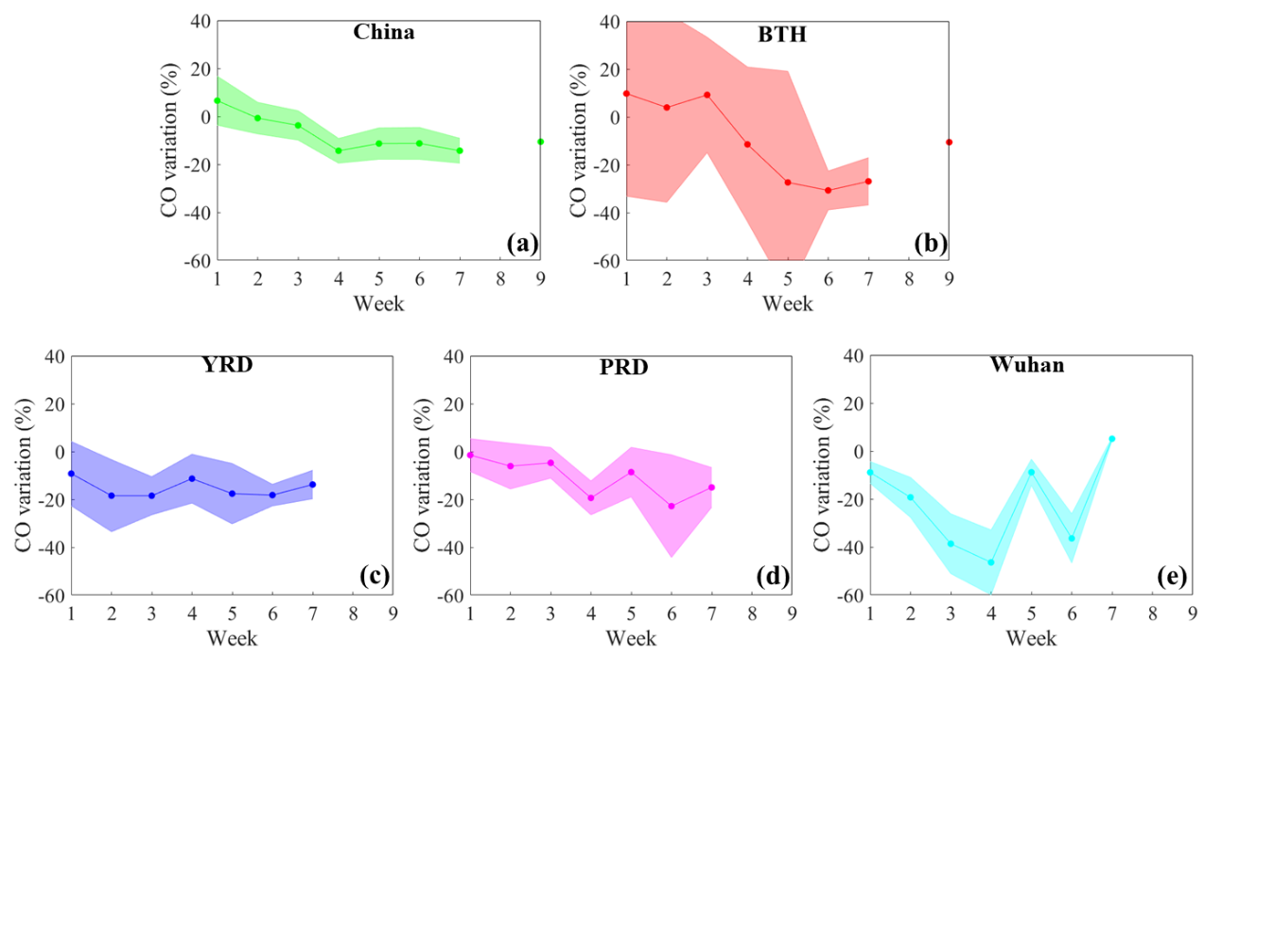
**

**Figure S13.** The ambient 8-h O_3_ variation ratios in China (a), BTH (b), YRD (c), PRD (d), and Wuhan (e) during the COVID-19 outbreak compared with the same period during 2018-2019.

**
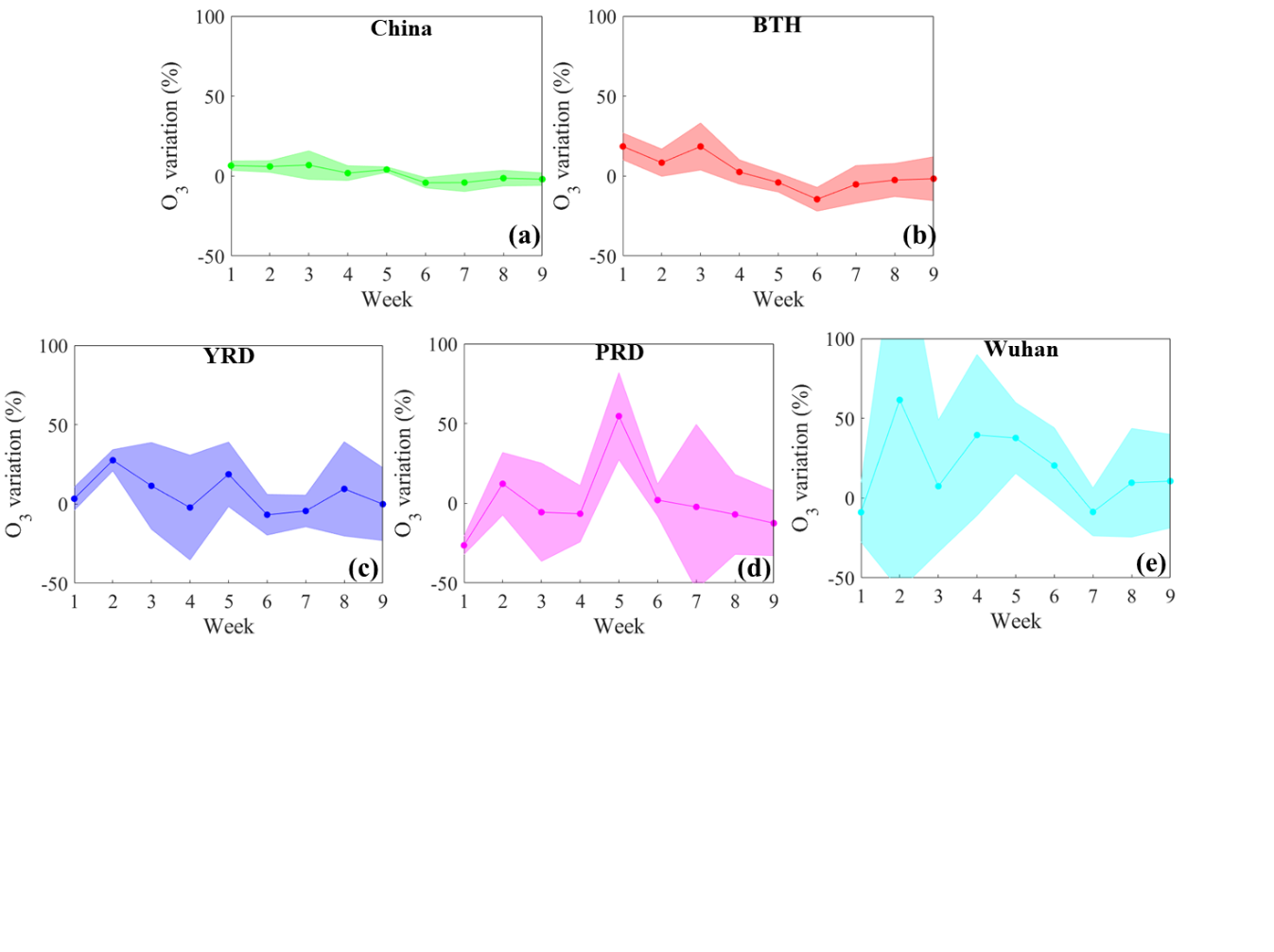
**

**Table S1.** The basic information and data sources of predictors for NO_2_ estimates in our study.

| Dataset | Variable | Unit | Spatial resolution | Time resolution | Data source |
| --- | --- | --- | --- | --- | --- |
| NO_2_ | NO_2_ | μg/m^3^ | -- | Daily | Environmental Protection Bureau |
| NO_2_ tropospheric column | NO_2_ column | mole/cm^2^ | 0.25° | Daily | NASA |
| Meteorology | D_2m_ | ℃ | 0.25° | 1-hour | ERA  reanalysis  product |
|  | T_2m_ | ℃ | 0.25° | 1-hour |  |
|  | U_10_ | m/s | 0.25° | 1-hour |  |
|  | V_10_ | m/s | 0.25° | 1-hour |  |
|  | E | mm | 0.25° | 1-hour |  |
|  | Mbld | W/m^2^ | 0.25° | 1-hour |  |
|  | Sp | hPa | 0.25° | 1-hour |  |
|  | Tp | mm | 0.25° | 1-hour |  |
| Land use types | Waters | m^2^ | 30 m | Annually | CRESDC |
|  | Grassland | m^2^ | 30 m | Annually |  |
|  | Urban | m^2^ | 30 m | Annually |  |
|  | Forest | m^2^ | 30 m | Annually |  |
|  | Agricultural land | m^2^ | 30 m | Annually |  |
| Elevation | DEM | m | 30 m | -- | SRTM |
| Socioeconomic data | Population | -- | 1 km | 5-yr | CRESDC |

**Table S2.** Cause-specific coefficients (β) and 95% confidence intervals of the concentration-response functions for daily NO_2_ concentration in our study. β denotes the percentage change of daily mortality associated with a 10 μg/m^3^ increase of daily NO_2_ [3].

| Health endpoints | NO_2_ (%) |
| --- | --- |
| All-cause | 0.90 (0.70-1.10) |
| Cardiovascular disease | 0.90 (0.70-1.20) |
| Respiratory disease | 1.20 (0.90-1.50) |
| COPD | 1.60 (1.10-2.00) |

**Table S3.** Cause-specific coefficients (β) and 95% confidence intervals of the concentration-response functions for 8-h O_3_ concentration in our study. β denotes the percentage change of daily mortality associated with a 10 μg/m^3^ increase of daily NO_2_[3].

| Health endpoints | 8-h O_3_ (%) |
| --- | --- |
| All-cause | 0.24 (0.13-0.35) |
| Cardiovascular disease | 0.27 (0.10-0.44) |
| Respiratory disease | 0.18 (-0.11-0.47) |
| COPD | 0.20 (-0.13-0.53) |

**Table S4. The baseline mortality used in the estimation of mortality (per 1000 people, 95% CI).**

| Health endpoints | Frequency (95% CI) | References |
| --- | --- | --- |
| All cause | Actual mortality rate | CSY (2015) |
| Cardiovascular disease | 0.990 | CMH (2015) |
| Respiratory disease | 0.756 | CMH (2015) |
| COPD | 0.880 | CMH (2015) |

**References**

1. CSY, 2015. Chinese Statistical Yearbook. China Statistics Bureau. (in Chinese)
2. CMH, 2015. Report of the Second National Health Service Survey. China Ministry of Health. (in Chinese)
3. Chen, R.; Yin, P.; Meng, X.; Wang, L.; Liu, C.; Niu, Y.; Lin, Z.; Liu, Y.; Liu, J.; Qi, J.; You, J.; Kan, H.; Zhou, M., Associations Between Ambient Nitrogen Dioxide and Daily Cause-specific Mortality: Evidence from 272 Chinese Cities. Epidemiology 2018, 29 (4), 482-489.
